# La(OH)_3_ nanoparticles immobilized on Fe_3_O_4_@chitosan composites as novel magnetic nanocatalysts for sonochemical oxidation of benzyl alcohol to benzaldehyde

**DOI:** 10.1039/d1ra05848g

**Published:** 2021-11-08

**Authors:** Fereshteh Javidfar, Manoochehr Fadaeian, Javad Safaei Ghomi

**Affiliations:** Department of Chemistry, Qom Branch, Islamic Azad University Post box: 37491-13191 Qom I. R. Iran fadaeian_m@yahoo.com +98 9128236206 +98 2537780045; Department of Organic Chemistry, Faculty of Chemistry, University of Kashan Kashan I. R. Iran

## Abstract

This work introduces an eco-friendly method for immobilization of La(OH)_3_ nanoparticles on modified Fe_3_O_4_ nanoparticles. The structural and morphological characteristics of the nanocatalyst were determined by various analytical techniques including, FT-IR, EDS, FESEM, VSM and XRD. The catalytic efficiency of the Fe_3_O_4_@Cs/La(OH)_3_ composite as a heterogeneous nanocatalyst was evaluated by selective oxidation of benzylic alcohols to aldehydes. The optimum reaction conditions including time, temperature, nanocatalyst dosage, and solvent were investigated for ultrasound-assisted oxidation processes. Furthermore, the magnetic nanocatalyst was recovered up to seven times without considerable activity loss. Furthermore, the proposed nanocomposite had a remarkable effect on reducing the reaction time and enhancing the yield.

## Abstract 2

A sustainable synthesis of novel Fe_3_O_4_@CS/La(OH)_3_ as a nanocatalyst for oxidation of benzyl alcohols to benzaldehydes has been developed.

## Graphical abstract

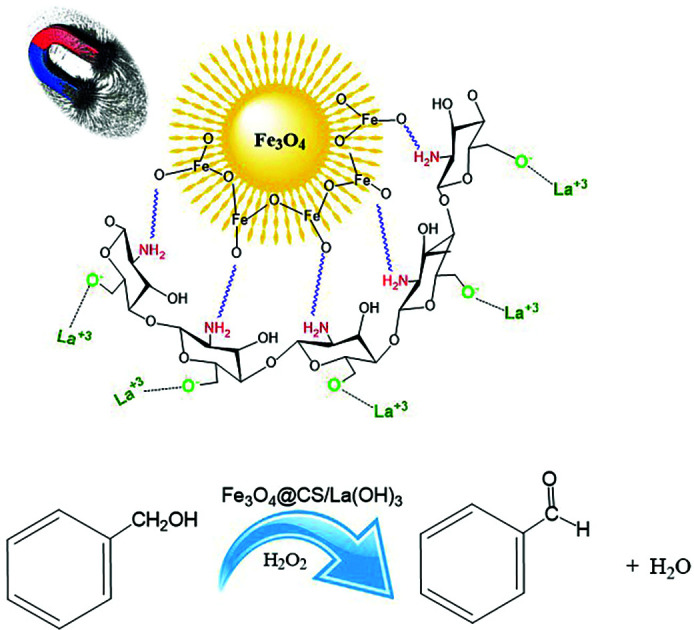

## 1. Introduction

Oxidation transformations have attracted much interest due to their potential applications and functionality in the chemical and materials industries. Among different oxidation reactions, oxidation of benzyl alcohols into the corresponding benzaldehydes is a prominent chemical transformation in organic chemistry.^1–5^ Aldehydes, which have various applications in different fields such as pharmaceuticals, dyes, perfumes, agriculture, food, beverages, agribusiness industries, and chemicals, are used as valuable oxygen-containing intermediates and raw materials in organic chemistry. In the past, despite numerous available methods for selective oxidation processes, most of them were not without drawbacks, generating a lot of by-products and pollutants. These processes require toxic, expensive, or hazardous chemicals (such as pyridinium chlorochromate (PCC), permanganate (MnO_4_^−^), dichromate (Cr_2_O_7_^2−^), chromium trioxide (CrO_3_)), as oxidants that lead to safety and ecological problems.^6,7^ Thus, the development of a new method for the construction of heterogeneous (nano)catalysts is a matter of increasing attention in the catalysis field. In recent years, biopolymer derived nanocatalysts have been considered as heterogeneous catalysts with excellent catalytic activity for chemical transformations, particularly, in oxidation reactions.

Among these, ecofriendly polysaccharides are used as efficient supports in the functionalization of metal nanoparticles.^5^

**Scheme 1:**
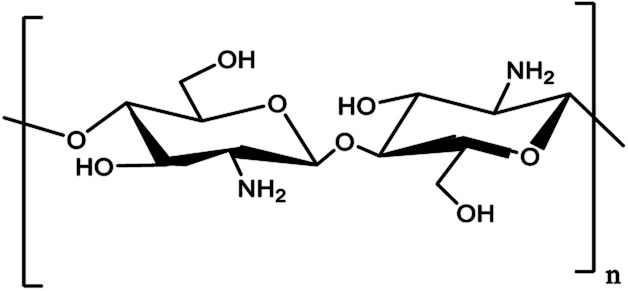
Chitosan structure.

Chitosan (CS) is the second most abundant biopolymer (after cellulose) on the earth which is applied in many heterogeneous catalytic systems. Utilization of chitosan as catalyst support has attracted profound attention due to its significant properties such as low cost, resource abundance, hydrophilicity, chemical stability, eco-friendliness, biodegradability, non-toxicity, significant thermal stability, and antioxidant properties.^8–11^ In addition, the presence of NH_2_ and OH functional groups produces appropriate arrangements such as chelating ligands to coordinate various metal ions^9^ (Scheme 1).

On the other hand, effective recycling and easy separation are important factors in developing heterogeneous catalysts.^5^ In the past few decades, increased use of Fe_3_O_4_ nanoparticles (NPs) in heterogeneous catalysts have captured intense attention owing to their unique catalytic properties such as super-paramagnetism, non-toxicity, easy preparation, chemical stability, easy and excellent recyclability, and reusability.^12,13^

The great properties of magnetic chitosan (Fe_3_O_4_@CS) have led to its use in different fields such as drug delivery systems, oxidation and sulfoxidation process, removal of heavy metals, *etc.*^5^

On the other hand, ultrasonic-engineered reactions are more effective than traditional approaches (conventional heating conditions).^5^ Ultrasound (US) irradiation can make changes in reactivity, increase modifications by improving surrender, reduce reaction time, and finally replace dangerous reagents with safe ones.^14,15^ Therefore, selective oxidation reactions using the nanomaterials in conjunction with US irradiation, can be highly efficient.^16^

In 1794, lanthanum oxide was discovered by Johann Gadolin.^17^ Among the rare earth oxides, lanthanum oxide has been considered as catalyst in various reactions due to its unique properties (good paramagnetic sensitivity, saturated magnetization, magnetostrictive properties, the large bandgap, *etc*…).^18^ Therefore, lanthanum(iii) oxide can be a good candidate for improvement of catalytic activity.^18,19^

With this background, we designed, prepared and characterized Fe_3_O_4_@CS/La(OH)_3_ nanocomposites as a novel heterogeneous catalyst for ultrasound-assisted oxidation reaction. Some of the strange and unique attributes of applied oxidation protocol are short reaction time, great yield, green condition, simple recovery of nano catalysts, and easy workup.

## 2. Results and discussion

**Fig. 1:**
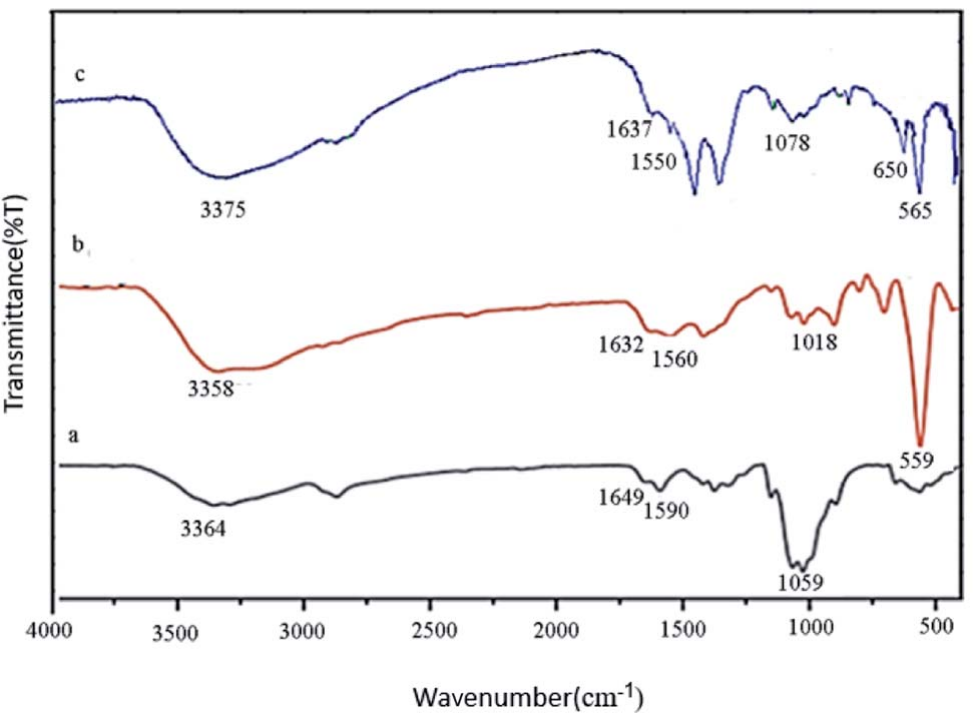
FT-IR spectra of (a) pure CS, (b) Fe_3_O_4_@CS, (c) Fe_3_O_4_@CS/La(OH)_3_ composites.

FT-IR spectroscopy is one of the most important techniques for identifying organic functional groups. The FT-IR spectra of Fe_3_O_4_@CS/La(OH)_3_, Fe_3_O_4_@CS and pure CS were shown in Fig. 1. As shown in Fig. 1a–c, the broad absorption band at 3364, 3358 and 3375 cm^−1^ belong to the amino and hydroxyl groups of chitosan. The bands at 1649, 1632 and 1637 cm^−1^ are related to the C=O stretching vibration of the amide group. The bending vibration of the amino group appeared at 1590, 1560 and 1550 cm^−1^. Also respectively, 1059, 1018, and 1078 cm^−1^ represented the C–O stretching vibration of C–OH of chitosan in Fig. 1a–c. As shown in Fig. 1b and c, the absorption band at 559 cm^−1^ (or 565 cm^−1^) belongs to the Fe–O stretching vibrations.^20^ The medium absorption band at 650 cm^−1^ was because of La–O stretching vibration (Fig. 1c).^17^

**Fig. 2:**
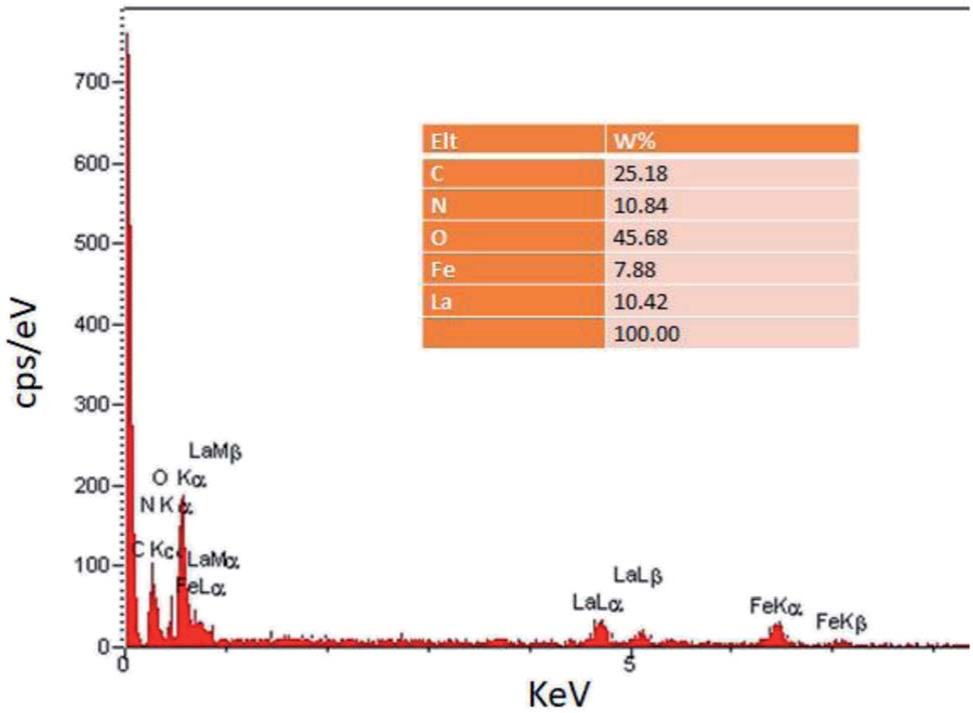
EDS spectrum of Fe_3_O_4_@CS/La(OH)_3_.

In the EDS spectrum (Fig. 2), the presence of all elements including C, N, O, Fe, and La, is determined according to the energy, which indicates the confirmation of product purity.

**Fig. 3:**
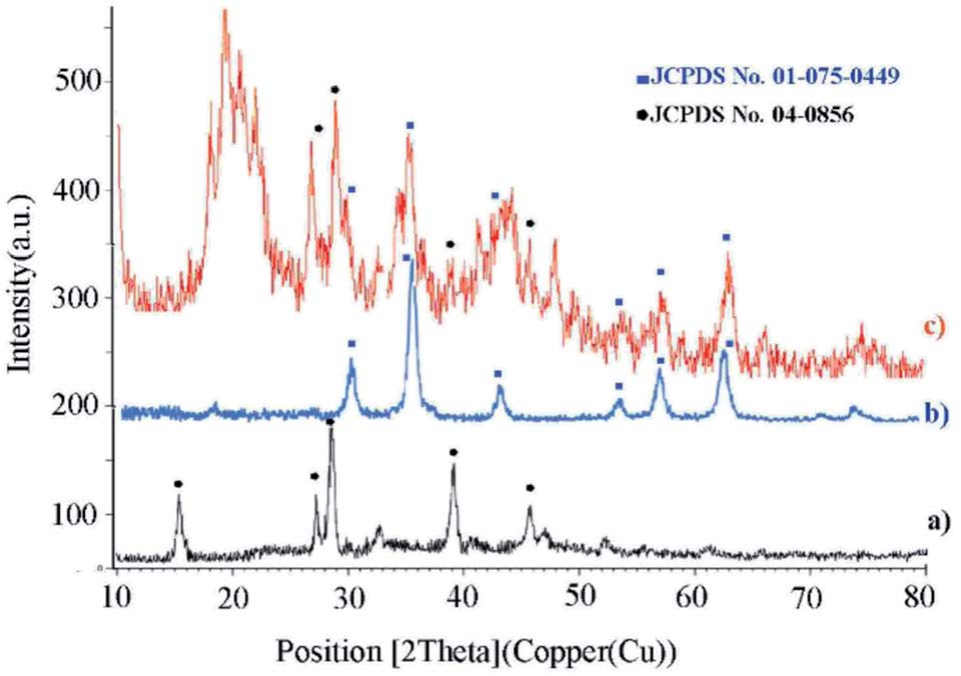
XRD patterns of (a) La(OH)_3_, (b) Fe_3_O_4_, and (c) Fe_3_O_4_@CS/La(OH)_3_.

XRD analysis determines a direct method for the structure of matter and fuzzy composition. This method can be used to determine lattice geometry, unknown materials, crystal size and phase, the lattice constant and defect, orientation of crystal monolayers, *etc.* Hence, it was used to identify the crystallite structure of Fe_3_O_4_@CS/La(OH)_3_ nanocatalyst. The XRD patterns for Fe_3_O_4_, La(OH)_3_ nanoparticles, and Fe_3_O_4_@CS/La(OH)_3_ nanocomposite are illustrated in Fig. 3a–c. Characteristic peaks for Fe_3_O_4_ are shown in the region at 2*θ* of 30.1°, 35.6611°, 44.2975°, 53.8058°, 57.3929°, and 62.9953° which correspond to (220), (311), (400), (422), (511), and (440) respectively (Fe_3_O_4_; JCPDS card no. 01-075-0449) in a good agreement with literature.^21^ The broad diffraction peaks that appeared around 2*θ* = 19° for Fe_3_O_4_@CS/La(OH)_3_ sample are related to chitosan (Fig. 3c).^22^ In addition, the XRD diffraction peaks are observed at 27.2912°, 28.3643°, 39.6213°, and 48.1237° are related to La_2_O_3_ which correspond to (222), (300), (400), and (622) respectively (La(OH)_3_; JCPDS card no. 04-0856).^17,18,23^ The observed peaks show that the structure of Fe_3_O_4_ and La(OH)_3_ have not changed during the composition process.

**Fig. 4:**
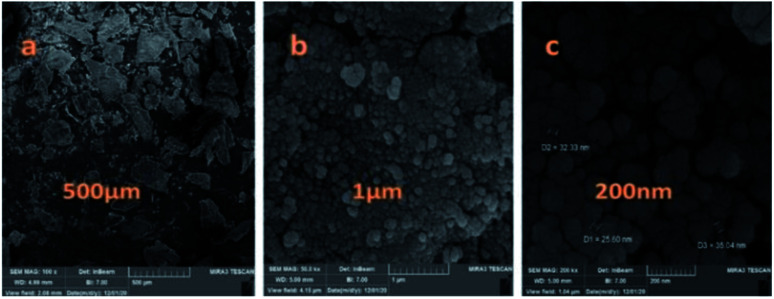
FE-SEM images of the Fe_3_O_4_@CS/La(OH)_3_ catalyst (a–c).

The particle size, surface properties, and shape of prepared nanocatalyst were observed using FESEM with various magnifications. The FESEM images of Fe_3_O_4_@CS/La(OH)_3_ nanocomposites show a uniform spherical shape with the average particle size about 28 nm (Fig. 4).

**Fig. 5:**
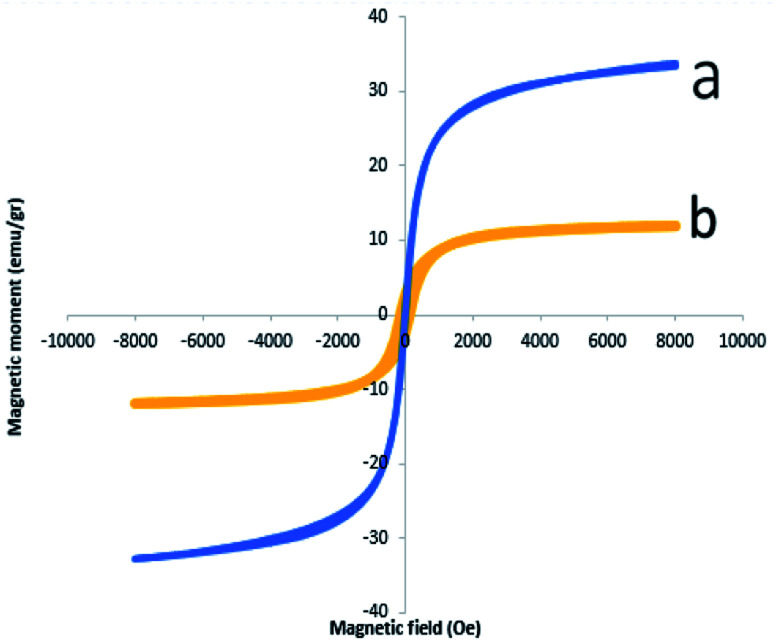
VSM (a) Fe_3_O_4_, (b) Fe_3_O_4_@CS/La(OH)_3_ nanocomposites.

Magnetic properties of Fe_3_O_4_ NPs and Fe_3_O_4_@CS/La(OH)_3_ composites were measured by VSM analysis (Fig. 5). The hysteresis loops of pure Fe_3_O_4_ NPs and Fe_3_O_4_@CS/La(OH)_3_ nanocomposites are S-like curves. Both samples have super paramagnetic behavior which facilitates magnetic separation. The specific saturation magnetization of the pure Fe_3_O_4_ NPs, and Fe_3_O_4_@CS/La(OH)_3_ composites were 33.74, and 11.95 emu g^−1^, respectively. Although the addition of CS layer and La(OH)_3_ nanoparticles on Fe_3_O_4_ surface led to decreased magnetic properties, Fe_3_O_4_@CS/La(OH)_3_ composites saturation magnetization was enough for a quick magnetic separation with an external magnet. The reason for the decreased saturation magnetization value for the Fe_3_O_4_@CS/La(OH)_3_ composite can be related to the presence of non-magnetic chitosan and the cover of CS/La(OH)_3_ hybrid materials shells on the magnetic Fe_3_O_4_ surface.^24^

### 2.1. Catalytic activity of Fe_3_O_4_@CS/La(OH)_3_ nanocomposites

Oxidation of benzyl alcohols to the corresponding aldehydes was performed under mild reaction conditions. Table 1 clearly shows the strength of Fe_3_O_4_@CS/La(OH)_3_ in the sonochemical oxidation process, and the catalytic performance of Fe_3_O_4_@CS/La(OH)_3_ was compared with different catalysts to investigate. Ultrasonic oxidation conditions play a key role in this process. Therefore, oxidation was done in the presence and absence of catalyst Fe_3_O_4_@CS/La(OH)_3_ (Table 1, entry 5–6). According to the test results, in the absence of the catalyst, the yield decreased (Table 1, entry 6). Sonication conditions had an outstanding role in this oxidation. Under silent conditions (classical heating/grinding/stirring) no significant yields were detected. According to the test results, a non-oxidizing and catalytic oxidation reaction may occur during the 50 mg test and the appropriate amount is to prepare 100% benzaldehyde in 5 minutes. Increasing the amount of catalyst causes the production of benzoic acid. In these experiments, catalyst Fe_3_O_4_@CS/La(OH)_3_ was compared with other catalysts (Table 1, entry 12–18). Finally, the catalyst with excellent results offers a very gentle and green option.

**Table 1:** Effect of different conditions on the benzyl alcohol oxidation^a^

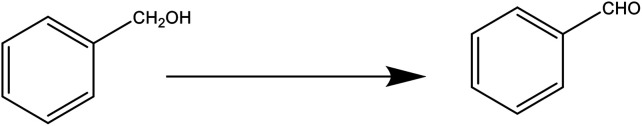							
Entry	Catalyst^ref.^	Catalyst (mg)	Time (min)	Temperature (°C)	Oxidant	Solvent	Yield^b^ (%)
1	Fe_3_O_4_@CS/La(OH)_3_	50	15	r.t/US	H_2_O_2_	*m*-Xylene	58
2	Fe_3_O_4_@CS/La(OH)_3_	50	15	r.t/US	H_2_O_2_	Toluene	63
3	Fe_3_O_4_@CS/La(OH)_3_	50	15	r.t/US	H_2_O_2_	Acetonitrile	80
4	Fe_3_O_4_@CS/La(OH)_3_	50	15	r.t/US	H_2_O_2_	Ethanol	85
**5**	**Fe**_**3**_ **O**_**4**_ **@CS/La(OH)**_**3**_	**50**	**5**	**r.t/US**	**H**_**2**_ **O**_**2**_	**Solvent free**	**100**
6	Fe_3_O_4_	50	5	r.t/US	H_2_O_2_	Solvent free	76
7	Fe_3_O_4_@CS	50	5	r.t/US	H_2_O_2_	Solvent free	88
8	Null	—	15	r.t/US	H_2_O_2_	H_2_O	50
9	Fe_3_O_4_@CS/La(OH)_3_	75	10	r.t/US	H_2_O_2_	H_2_O	86
10	Fe_3_O_4_@CS/La(OH)_3_	50	180	130	H_2_O_2_	*m*-Xylene	87
11	Fe_3_O_4_@CS/La(OH)_3_	50	160	130	H_2_O_2_	Ethanol	88
12	Fe_3_O_4_@CS/La(OH)_3_	50	300	130	H_2_O_2_	Acetonitrile	87
13	Null	—	240	130	H_2_O_2_	*m*-Xylene	91
14	ZPCu^27^	0.005	60	90	H_2_O_2_	Solvent free	90
15	Au–Pd/C^28^	2	240	80	H_2_O_2_	Solvent free	11.32
16	ZnBr_2_ (ref. 29)	0.02	90	Reflux	Chloramine-T	CH_3_CN	96
17	Au/Al_2_O_3_ (ref. 30)	48	15	100	O_2_	Toluene	86
18	(TEAH)H_2_PW_12_O_40_ (ref. 31)	0.04	180	100	H_2_O_2_	H_2_O	99.6
19	FSPC^32^	50	15	r.t	H_2_O_2_	Acetonitrile	70
20	WO_4_@PMO-IL^33^	0.0015	720	90	H_2_O_2_	CH_3_CN:H_2_O	75

a Reaction conditions: benzyl alcohols (1 mmol), H_2_O_2_ (1 ml), Fe_3_O_4_@CS/La(OH)_3_ (0.05 g).

b Isolated yield.

**Scheme 2:**
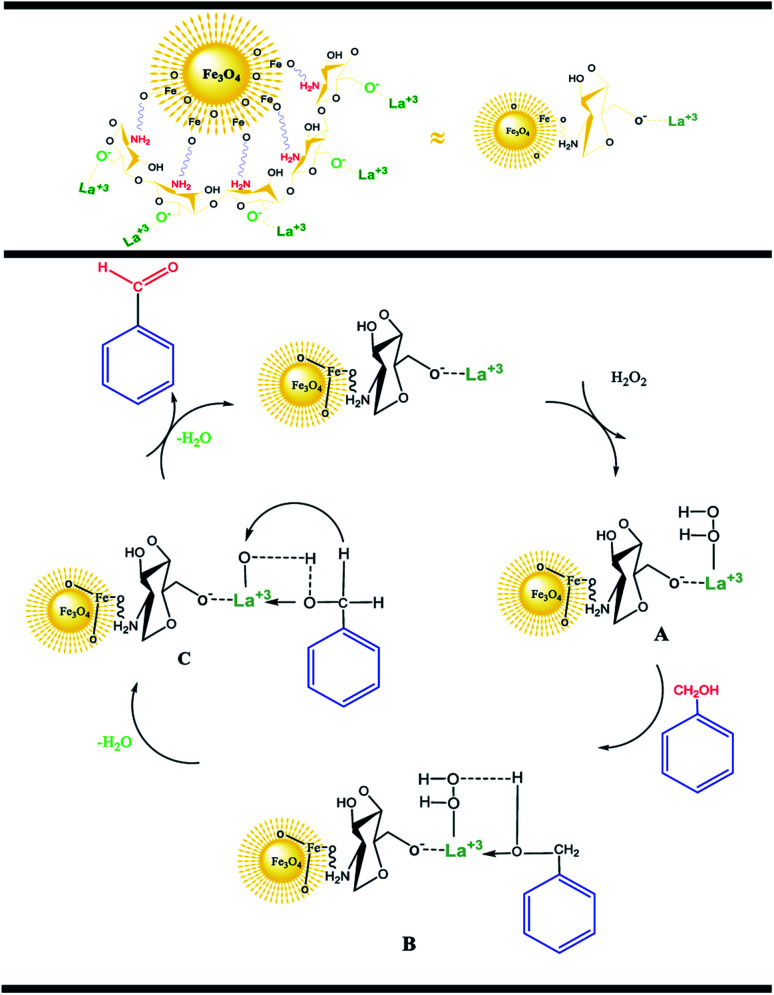
A possible reaction mechanism.

According to the obtained results, a wide range of benzyl alcohols bearing either electron-donating or electron-withdrawing groups were successfully converted to benzaldehyde in short reaction times using Fe_3_O_4_@CS/La(OH)_3_ (Table 2). Corresponding products of both groups were achieved without any over-oxidation (Table 2, entry 1–15). Steric hindrance in *ortho* and *meta* position decreased the reaction yields during longer reaction time (Table 2, entry 1, 4, 6, 8, 9 and 11) (Scheme 2).

**Table 2:** Oxidation of benzyl alcohols to benzyl aldehydes^a^

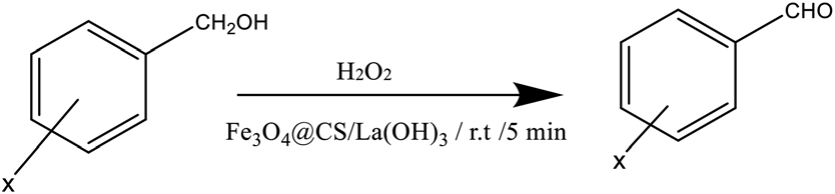				
Entry	X	Time (min)	Yield^b^ (%)	Selectivity (%)
1	3-Hydroxy	10	87	>99
2	4-Hydroxy	5	100	100
3	4-Chloro	5	99	100
4	2-Chloro	15	87	>99
5	4-Methoxy	5	96	100
6	3-Methoxy	10	90	>99
7	4-Methyl	5	96	100
8	3-Methyl	10	81	>99
9	3-iPr	10	87	>99
10	4-Nitro	5	96	100
11	3-Nitro	10	87	>99
12	4-Fluoro	5	97	>99
13	4-Bromo	5	98	>99
14	3-Bromo	10	83	>99
15	H	5	100	100

a Reaction conditions: benzyl alcohols (1 mmol), H_2_O_2_ (1 ml), Fe_3_O_4_@CS/La(OH)_3_ (0.05 g).

b Isolated yield.

According to the research, an acceptable mechanism for this oxidation has been designed. The results illustrated that La^3+^ acts as Lewis acid site in the oxidation reaction of benzylic alcohols. At first, the La^3+^ was coordinated to the O of H_2_O_2_ and generated intermediate A. After that, intermediate A reacted with benzyl alcohols to create intermediate B. The elimination of an H_2_O molecule from intermediate B resulted in intermediate C. Finally, the removal of the second H_2_O molecule provided the desired benzaldehyde.^25,26^

### 2.2. Catalyst reutilization

**Fig. 6:**
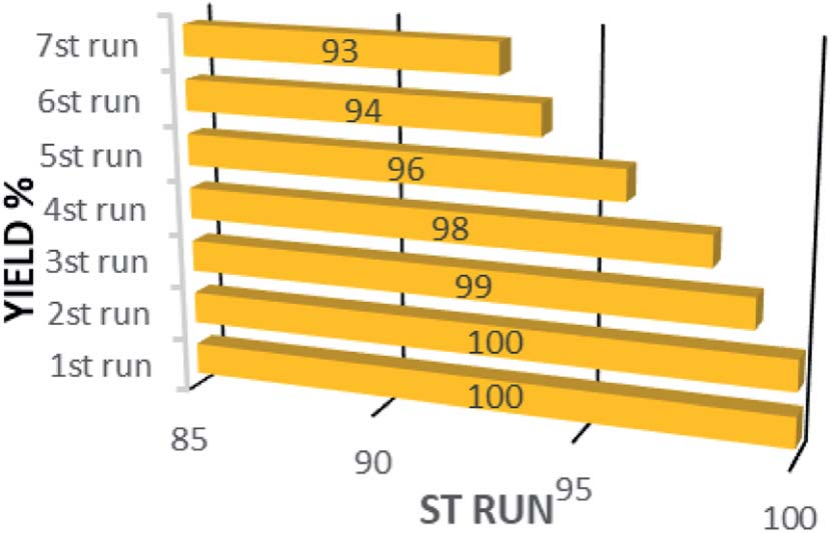
Recyclability of sonocatalyst.

In addition to the catalytic activity, the stability plays a vital role in catalysis field. In this work, the reusability of the catalyst was tested under optimum reaction conditions. The results show that the Fe_3_O_4_@CS/La(OH)_3_ successfully recovered up to 7th cycle (Fig. 6).

**Fig. 7:**
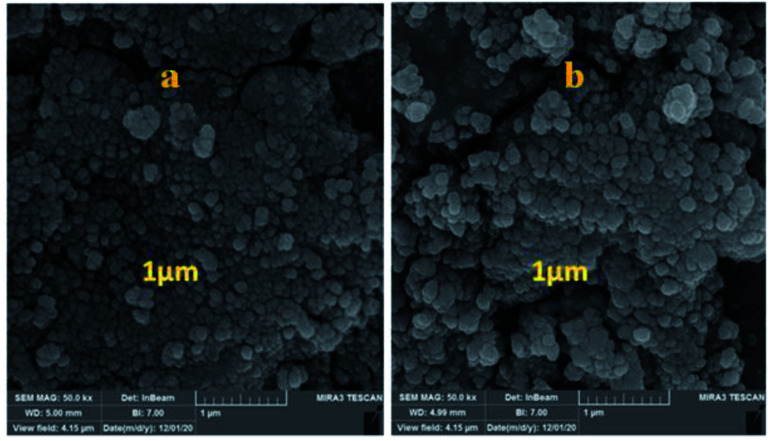
Comparison of FE-SEM of the prepared Fe_3_O_4_@CS/La(OH)_3_ (a) before and (b) after 7 runs.

The morphology of the Fe_3_O_4_@CS/La(OH)_3_ nanocatalyst after 7 reuse periods is shown in Fig. 7b. The spherical morphology of Fe_3_O_4_@CS/La(OH)_3_ is preserved, indicating that the nanocatalyst was well stable.

**Fig. 8:**
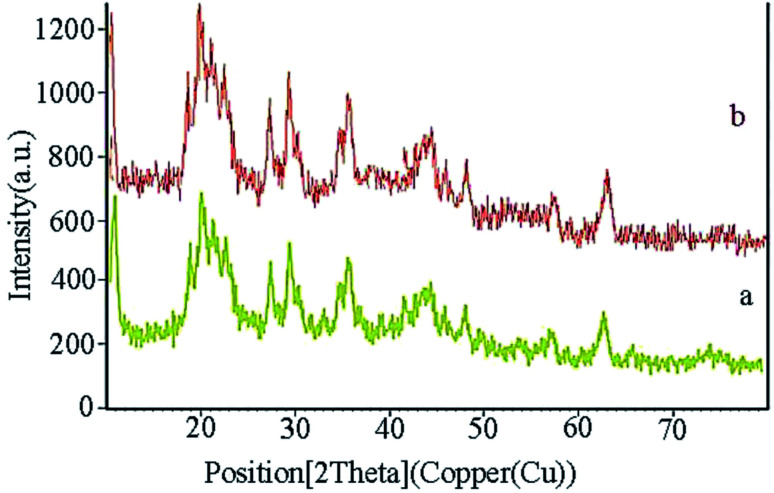
Comparison of XRD of the prepared Fe_3_O_4_@CS/La(OH)_3_ (a) before and (b) after 7 runs.

XRD of the Fe_3_O_4_@CS/La(OH)_3_ nanocatalyst after 7 reuse periods is shown in Fig. 8b. Characteristic peaks for Fe_3_O_4_@CS/La(OH)_3_ are preserved, indicating that the nanocatalyst was well stable and pure.

## 3. Experimental

### 3.1. Chemicals and apparatus

In this project, all the chemicals, including alcohol and solvents required for the tests, were purchased from Merck and Aldrich. FT-IR samples were collected by KBr pellets and their spectra were detected by PerkinElmer 1600 FTIR spectrometer. The morphology and size of the samples were determined by scanning electron microscopy (SEM) and the crystals were formed by X-ray diffraction (XRD) and scattered X-ray energy spectroscopy (EDX) and vibrating sample magnetometer (VSM). The oxidation products were examined by gas chromatographic spectrometry (GC).

### 3.2. Preparation of Fe_3_O_4_ nanoparticles

Fe_3_O_4_ magnetic nanoparticles (MNPs) were constructed by the chemical co-precipitation method.^34^ Approximately 1.7 g of Fe(ii) and 4.75 g of Fe(iii) salts were dissolved in deionized water (200 ml). The mixture was stirred at 60 °C under N_2_ atmosphere, then 7.5 ml of NH_3_ solution was added. Then the mixture of reaction was allowed to occur for 1 h at 60 °C. Finally, the dark solid was magnetically separated, washed with ionized water, and dried at 60 °C overnight. In particular, to avoid the conversion of Fe_3_O_4_ to Fe_2_O_3_ in air, all of the synthetic procedure was conducted under N_2_ atmosphere.

### 3.3. Preparation of Fe_3_O_4_@CS

First, 0.01 g of chitosan was dissolved in 10 ml of ethanoic acid. Subsequently, about 0.25 g of Fe_3_O_4_ was added to the chitosan solution and dispersed for half an hour. The resulting solution was mechanically stirred at 60 °C. Next, solution (prepared by dissolving 0.02 g of STPP (sodium tripolyphosphate) in 50 ml of deionized water) was dropwise added at a rate of 4.5 ml h^−1^. At this stage, the ionic gelation of chitosan was created on the Fe_3_O_4_ MNPs surface. After filtering, the product was dried for 36 hours at −20 °C. Then, the core–shell product of Fe_3_O_4_@CS nanoparticles was obtained.^34^

### 3.4. Procedure for the preparation of Fe_3_O_4_@CS/La(OH)_3_

Fe_3_O_4_@CS/La(OH)_3_ was generated by dispersing 0.1 g of Fe_3_O_4_@CS in deionized water (50 ml) for 1 hour. Next, 0.05 g of LaCl_3_·7H_2_O was added. The whole mixture was stirred about 2 hours under reflux condition. The synthesized nanocomposites were collected by an external magnet and were washed with distilled water.

### 3.5. General procedure for oxidation of benzyl alcohols

Benzyl alcohol oxidation and synthesized catalyst were investigated. Benzyl alcohol (1 mmol), nanocatalyst Fe_3_O_4_@CS/La(OH)_3_ (50 mg), and H_2_O_2_ (1 ml) were sonicated at 25 °C. After completion of the oxidation process, the catalyst was separated using a magnet. Then, the organic phase was extracted with EtOAc, and the products were investigated through GC analysis.^35^

## 4. Conclusions

In summary, we designed and fabricated a novel magnetic nanostructure of Fe_3_O_4_@CS/La(OH)_3_ for the oxidation of different types of benzyl alcohols to benzaldehyde under green conditions for the first time. Accordingly, the utilization of Fe_3_O_4_@CS/La(OH)_3_ as a nanocatalyst can not only decrease the reaction time, but also increase the selectivity and yields. The presence of Fe_3_O_4_@CS/La(OH)_3_ showed outstanding catalytic performance with high to excellent conversions for different substituted benzylic alcohols and selectivity for benzyl alcohol at room temperature under US conditions, short reaction time, inexpensive and excellent conversion yields according to the green chemistry principles. Ultrasound irradiation process oxidation of benzyl alcohols with high selectivity is a more effective manner than the conventional heating method due to the synergistic effects between the ultrasound radiation, H_2_O_2_, and the Fe_3_O_4_@CS/La(OH)_3_ nanocatalyst. The morphology of the Fe_3_O_4_@CS/La(OH)_3_ nanocatalyst confirmed that after 7 reuse periods, nanocatalyst was well stable and did not reveal a significant difference.

## Conflicts of interest

The authors stated that they had no financial or personal interest in preparing the material reported in this article.

## Supplementary Material
